# Surgical treatment of double aortic arch in infants

**DOI:** 10.3389/fped.2025.1622525

**Published:** 2025-08-14

**Authors:** Chenhan Wang, Bingjie Chen, Jingnan Chen, Jinwen Luo, Guangxian Yang, Liwen Yi, Xicheng Deng

**Affiliations:** Department of Cardiothoracic Surgery, The Affiliated Children’s Hospital of Xiangya School of Medicine, Central South University (Hunan Children's Hospital), Changsha, Hunan, China

**Keywords:** double aortic arch, infants, surgical treatment, cardiopulmonary bypass, follow-up

## Abstract

**Background:**

Double aortic arch (DAA) is a rare congenital vascular anomaly resulting in a complete vascular ring that encircles and compresses the trachea and esophagus, leading to respiratory and gastrointestinal symptoms. Accurate diagnosis and timely surgical intervention are essential for symptom relief and preventing complications. However, data on surgical outcomes and long-term follow-up are limited. This study retrospectively analyzed surgical outcomes and perioperative management of DAA to elucidate optimal diagnostic and therapeutic approaches.

**Method:**

A retrospective cohort study enrolled patients undergoing DAA repair (August 2015–May 2024). Participants were stratified into isolated DAA or DAA with associated intracardiac anomalies groups. Demographic, operative, and outcome variables were compared.

**Results:**

Among 10 patients undergoing double aortic arch repair, 6 comprised the isolated DAA group (3 males/3 females; mean age 3.70 ± 3.18 months; mean weight 6.28 ± 2.77 kg) and 4 had associated intracardiac anomalies (3 males/1 female; mean age 6.70 ± 6.12 months; mean weight 6.15 ± 3.59 kg). Isolated DAA patients and those with intracardiac anomalies showed no statistically significant differences in: symptom onset (28.17 ± 37.66d vs. 30.50 ± 41.96d), anatomic subtypes (dominant right arch 83% vs. 50%), extracardiac anomaly rates (50% vs. 75%), or clinical manifestations—respiratory (67% vs. 100%) and other systemic (17% vs. 75%) (all *P* > 0.05). All procedures were successfully completed with significantly shorter operative time in the isolated group (104.83 ± 22.23 vs. 233.25 ± 38.55 min, *P* < 0.001). No significant intergroup differences (*P* > 0.05) were observed in preoperative ventilation, blood loss, ventilator duration, Cardiac Intensive Care Unit stay, drainage duration, hospitalization, or complication rates. During mean 12.7-month follow-up (1–36 months), near-complete symptom resolution occurred in 9/10 survivors, with one death from respiratory failure in a comorbid patient 10 days post-discharge.

**Conclusion:**

Surgical repair of double aortic arch demonstrates acceptable safety and efficacy in both infants and children, with favorable short-to-midterm clinical outcomes regardless of concomitant intracardiac anomalies.

## 1 Introduction

Double aortic arch (DAA) is a rare congenital cardiovascular anomaly affecting approximately 0.05% to 0.30% of all individuals with congenital heart diseases (1). DAA arises during embryonic development due to abnormal development of the aortic arch complex, when bilateral fourth branchial arches persist (2). After arising from the ascending aorta (AAO), the aortic arch bifurcates into a left aortic arch (LAA) and a right aortic arch (RAA). The LAA and RAA respectively course anteriorly and posteriorly to the trachea and esophagus and converge at the origin of the descending aorta (DAO), forming a complete vascular ring encircling the trachea and esophagus, which causes varying degrees of compression (3). As children age, these compressive symptoms become more pronounced, profoundly compromising somatic development and quality of life, necessitating timely diagnosis and definitive surgical repair.

This study analyzed the diagnostic approach, surgical management, and short- and medium-term clinical outcomes for patients with DAA to provide a reference that can assist clinicians in managing similar cases.

## 2 Materials and methods

This is a single-center study. Between August 2015 and May 2024, all patients who underwent surgical repair for DAA at the Affiliated Children's Hospital of Xiangya School of Medicine, Central South University (Hunan Children's Hospital) were included in the study. All patients were diagnosed with DAA preoperatively using echocardiography and computed tomography angiography (CTA), in order to define DAA anatomical subtypes and identify the positions of the dominant and nondominant aortic arches. Written informed consent was obtained from all parents or legal guardians for minors. After approval was granted from the hospital's ethics committee (No.HCHLL-2024-309), a retrospective chart review was conducted to collect relevant data. Follow-up data were obtained through outpatient visits and telephone interviews.

Patients were categorized into the isolated DAA group and the DAA with associated intracardiac anomalies group according to the presence of intracardiac defects. Demographic, operative, and outcome variables were compared between groups. Statistical analysis was performed using SPSS 26.0 software. Continuous variables were expressed as mean ± standard deviation (mean ± SD), with intergroup comparisons performed using *t*-test. Categorical variables were presented as frequencies and percentages [*n* (%)], compared between groups by Fisher's exact test. A threshold of *P* ≤ 0.05 was considered statistically significant.

## 3 Results

### 3.1 Sociodemographic characteristics and preoperative data

The isolated DAA group comprised 6 patients (3 males, 3 females), and the DAA with associated intracardiac anomalies group included 4 patients (3 males, 1 female). Mean age at surgery was 3.70 ± 3.18 months for isolated DAA vs. 6.70 ± 6.12 months for DAA with associated intracardiac anomalies group. Mean weights measured 6.28 ± 2.77 kg (isolated DAA group) and 6.15 ± 3.59 kg (DAA with associated intracardiac anomalies group). No significant intergroup differences (*P* > 0.05) were observed in sex distribution, age, or weight. In the isolated DAA group, symptom onset occurred at 28.17 ± 37.66 days, compared with 30.50 ± 41.96 days in the DAA with associated intracardiac anomalies group, showing no significant intergroup difference (*t* = −0.092, *P* = 0.929). Anatomic subtypes analysis revealed that the isolated DAA group was predominantly characterized by a dominant right arch (5 cases, 83%), with only one case (17%) exhibiting balanced arches. Conversely, the DAA with intracardiac anomalies group demonstrated an equal distribution of balanced arches and dominant right arches (2 cases each, 50%). This intergroup distribution did not reach statistical significance (*P* = 0.500). Extracardiac anomalies were present in 50% (3/6) of patients in the isolated group and 75% (3/4) of the DAA with intracardiac anomalies group, with no significant intergroup difference (*P* = 0.571). The extracardiac anomalies encompassed airway abnormalities (tracheal stenosis, laryngomalacia), gastrointestinal defects (esophageal stenosis), and multisystem disorders (thalassemia, malnutrition, hydrocele). Respiratory manifestations occurred in 100% (4/4) of patients in the DAA with associated intracardiac anomalies group, higher than the 67% (4/6) incidence observed in the isolated DAA group, though this difference did not reach statistical significance (*P* = 0.467). Similarly, despite lacking statistical significance, other systemic manifestations were more prevalent in the DAA with associated intracardiac anomalies group (75% vs. 17% in the isolated group, *P* = 0.190), predominantly comprising cardiovascular presentations (cyanosis after crying or activity, acute heart failure) and gastrointestinal manifestations (feeding difficulties) (Table 1).

**Table 1:** Comparison of sociodemographic characteristics and preoperative data between groups.

Characteristics	isolated DAA group (*n* = 6) mean ± SD/*n* (%)	DAA with associated intracardiac anomalies group (*n* = 4) mean ± SD/*n* (%)	*t*	*P*
Time of symptom onset (d)	28.17 ± 37.66	30.50 ± 41.96	−0.092	0.929
Male	3 (50%)	3 (75%)	—	0.571
Anatomy subtype^a^			—	0.500
Balanced arches	1 (17%)	2 (50%)		
Dominant right arch	5 (83%)	2 (50%)		
Associated extracardiac anomalies(Yes)^ab^	3 (50%)	3 (75%)	—	0.571
Respiratory manifestations(Yes)^a^	4 (67%)	4	—	0.467
Other systemic manifestations(Yes)^ac^	1 (17%)	3 (75%)	—	0.190
Preoperative mechanical ventilation (Yes)^a^	0	2 (50%)	—	0.133

^a^ Fisher's exact test.

^b^ The extracardiac anomalies encompassed airway abnormalities (tracheal stenosis, laryngomalacia), gastrointestinal defects (esophageal stenosis), and multisystem disorders (thalassemia, malnutrition, hydrocele).

^c^ Other systemic manifestations include cardiovascular presentations (cyanosis after crying or activity, acute heart failure) and gastrointestinal manifestations (feeding difficulties).

### 3.2 Surgical techniques

The surgical approach for treating DAA was determined on the basis of each patient's vascular anatomy. Isolated DAA was corrected without cardiopulmonary bypass (CPB) by using a posterolateral incision on the contralateral chest. Patients with associated intracardiac anomalies underwent CPB through a median sternotomy. The isolated DAA group had significantly shorter operative time than the DAA with intracardiac anomalies group (104.83 ± 22.23 min vs. 233.25 ± 38.55 min; *P* < 0.001). No significant difference in intraoperative blood loss was observed between groups (10.83 ± 3.76 ml vs. 17.50 ± 9.57 ml; *P* > 0.05). For cases requiring CPB, the mean bypass time was 152.8 ± 41.8 min and the mean aortic occlusion time was 75.5 ± 18.7 min.

#### 3.2.1 Isolated DAA

**Figure 1:**
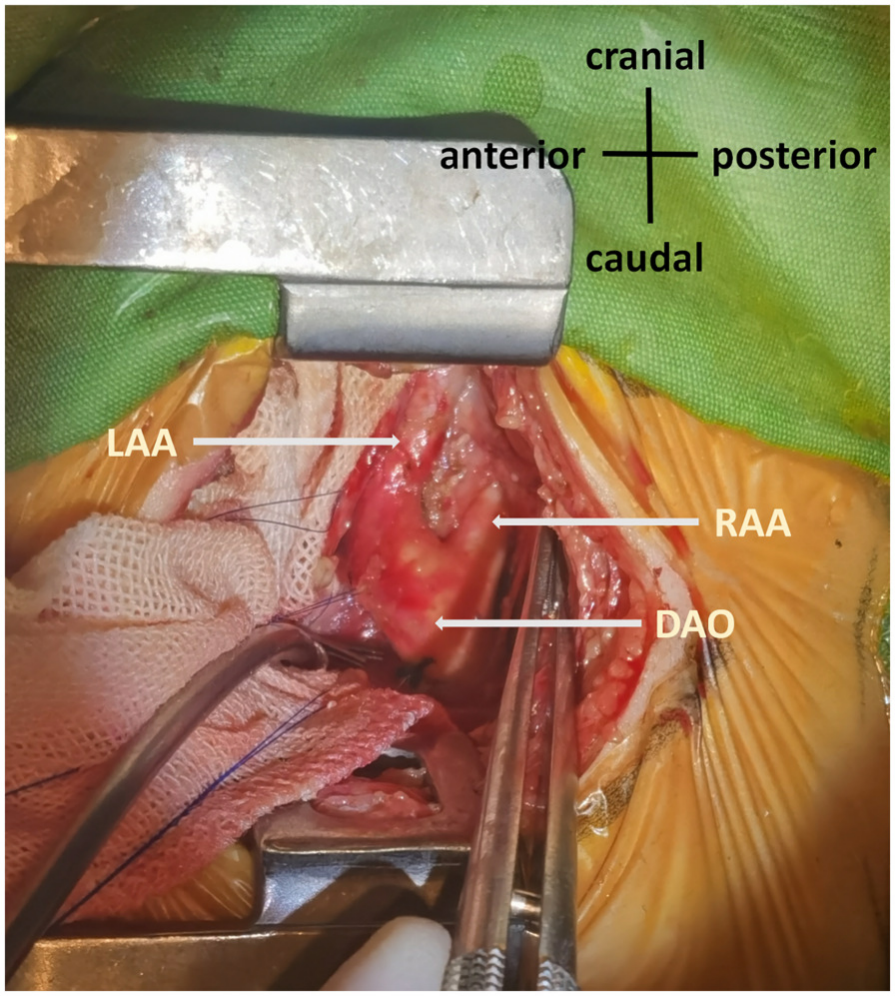
Double aortic arch. The left posterolateral thoracic approach revealed symmetrical development of the RAA and LAA. RAA, right aortic arch; LAA, left aortic arch; DAO, descending aorta.

Patients with a dominant right aortic arch were positioned in the right lateral decubitus position (Figure 1). Single-lung ventilation was performed using an Arndt end bronchial blocker, allowing the lung on the operative side to collapse, which provides better exposure of the surgical field and facilitates the surgeon's operation. After the chest was entered, the left upper lung was retracted, and the pleura was incised longitudinally and retracted with stay sutures. The ductus arteriosus or ligamentum ductus arteriosus was then dissected and ligated and further dissected upward to the LAA, including its branches—that is, the left subclavian artery and left common carotid artery. Dissection was continued downward to the confluence of the LAA and RAA. The distal LAA was clamped, with minimal impact on lower limb blood pressure, and was divided between clamps. After transecting the connection between the descending aorta and the hypoplastic arch, we performed suspension and fixation of the divided ends. This maneuver achieves dual objectives—immediate decompression and prevention of future diverticulum formation that could cause cicatricial tracheal compression. The tissues surrounding the esophagus and airway were completely dissected, and the mediastinal pleura was left unsutured. After adequate hemostasis was reached, a chest tube was inserted, and the thoracic incision was closed layer by layer, with tissue adhesive employed for skin closure. Patients were then transferred to the cardiac surgery intensive care unit (CICU).

#### 3.2.2 DAA with associated intracardiac anomalies

The patients with DAA combined with intracardiac anomalies such as atrial septal defect (ASD), ventricular septal defect (VSD) or tetralogy of Fallot (TOF) underwent CPB under general anesthesia and endotracheal intubation, this process does not require the use of single-lung ventilation. Each patient was placed in a supine position, and a midline incision was made using an electric saw to longitudinally open the sternum, exposing the pericardium. The right side of the pericardium was incised in the shape of “丄” and suspended. The ductus arteriosus or ligamentum ductus arteriosus was dissected, ligated, and sutured after division. The dissection was continued to the AAO, where RAA predominance was observed. The distal LAA was clamped and divided, after which the residual end was sutured and suspended. Following heparinization, CPB was established. CPB commenced at activated clotting time of >480 s following the clamping of the superior and inferior vena cava and AAO. After repairing the intracardiac defects, the sternum was then closed using continuous polydioxanone sutures. Postoperative management adhered to the institution's established protocol for lateral thoracotomy.

### 3.3 Postoperative complications

Both groups underwent successful surgical repair without in-hospital or operative mortality. Postoperative complications predominantly affected the respiratory system, with pulmonary infections occurring in three patients: one in the isolated DAA group and two in the DAA with associated intracardiac anomalies group. Of the latter group, one patient required reintubation and developed right vocal cord paralysis, which was improved by neuromuscular therapy. No significant differences (*P* > 0.05) were found between groups regarding mechanical ventilation duration, CICU stay duration, drainage tube retention duration, or postoperative length of stay (Table 2).

**Table 2:** Clinical outcomes between the two groups.

Characteristics	isolated DAA group (*n* = 6) mean ± SD/*n* (%)	DAA with associated intracardiac anomalies group (*n* = 4) mean ± SD/*n* (%)	*t*	*P*
Age at surgery (months)	3.70 ± 3.18	6.70 ± 6.12	−1.030	0.333
Weight at surgery (Kg)	6.28 ± 2.77	6.15 ± 3.59	0.067	0.949
Operation time (min)	104.83 ± 22.23	233.25 ± 38.55	−6.760	<0.001
Intraoperative blood loss (ml)	10.83 ± 3.76	17.50 ± 9.57	−1.571	0.155
Mechanical ventilation duration (h)	5.13 ± 2.97	25.21 ± 30.17	−1.670	0.133
CICU duration (h)	112.86 ± 27.29	426.21 ± 564.27	−1.769	0.115
Drainage tube retention duration (d)	4.83 ± 2.37	4.27 ± 1.18	0.425	0.682
Postoperative length of stay (d)	9.00 ± 1.41	24.00 ± 21.37	−1.769	0.115
Postoperative complications (Present)^a^	1 (17%)	2 (50%)	—	0.500

^a^ Fisher's exact test.

CICU, cardiac intensive care unit.

### 3.4 Follow-up

All patients completed the follow-up, with a follow-up duration of (12.7 ± 12.2) months. During the follow-up period, one patient died. This premature infant (gestational age 36 weeks + 4 days) weighing 2.8 kg, diagnosed with DAA combined with TOF and PDA, as well as moderate to severe malnutrition. Preoperatively, this patient already had severe myocardial damage (NT-proBNP: 15,808 pg/ml) and significant laryngomalacia and tracheal stenosis. Post-surgery, the patient was admitted to the cardiac intensive care unit. We attempted to extubate the endotracheal tube three times, however, due to the inability to maintain blood oxygen saturation after extubation, re-intubation was performed. After 53 days of treatment, the patient's family chose to discontinue treatment and leave the hospital. Tragically, 10 days after leaving the hospital, the patient died of respiratory failure secondary to persistent laryngomalacia.

## 4 Discussion

Approximately 80%, 10%, and 10% of DAA cases exhibit dominant right, dominant left, and balanced arches, respectively (4). In the present study, 70% of the patients had a dominant right, and 30% had balanced arches, with no dominant left cases, potentially due to regional genetic homogeneity or the limited sample size. While previous studies have predominantly focused on the treatment outcomes of isolated DAA (3, 5), this study stratified patients into an isolated DAA group and a DAA with associated intracardiac anomalies group, comparing their perioperative data and prognoses. Our comparison revealed that the operation time in the isolated DAA group was significantly shorter than that in the DAA with associated intracardiac anomalies group (104.83 ± 22.23 vs. 233.25 ± 38.55 min, *P* < 0.001). However, no significant differences (*P* > 0.05) were observed between the two groups in other key parameters, including preoperative mechanical ventilation, intraoperative blood loss, mechanical ventilation duration, CICU duration, drainage tube retention duration, postoperative length of stay, and postoperative complications. This finding indicates that although concomitant intracardiac anomalies increase surgical complexity and perioperative management difficulty, with appropriate management, the prognosis for DAA patients with associated intracardiac anomalies is comparable to that of patients with isolated DAA. This underscores the necessity and significant clinical value of implementing classification in the clinical diagnosis and treatment pathway for DAA.

The clinical presentation of DAA varies depending on the size and position of the arches and the degree of esophageal or tracheal compression (6). Tracheal compression can lead to coughing, wheezing, stridor, recurrent respiratory infections, and dyspnea (7, 8), with severe cases progressing to respiratory failure. In our cohort, two patients required rescue interventions due to respiratory arrest, consistent with previously reported severe presentations. Esophageal compression can result in dysphagia, vomiting, and developmental delays (8). Only one patient in this study was asymptomatic, likely due to the referral bias of our cardiac center, which primarily evaluates high-risk cases. Compared with prior studies reporting a mix of asymptomatic and symptomatic cases, our findings may overrepresent severe presentations due to the center's specialized patient population.

Regarding the impact of CPB in DAA cases with associated intracardiac anomalies, we contend that CPB is an essential intervention for managing such complex cases. It establishes a stable, bloodless surgical field, creating optimal conditions for intracardiac structural repair. However, a potential association exists between CPB utilization and prolonged postoperative mechanical ventilation duration: In this study, the mean mechanical ventilation duration was higher in the group with associated anomalies compared to the isolated DAA group (25.21 ± 30.17 h vs. 5.13 ± 2.97 h), although the intergroup difference did not reach statistical significance (*P* > 0.133). This prolongation may be closely linked to the systemic inflammatory response syndrome induced by CPB—contact between blood and artificial materials activates cascades involving complement, coagulation, and inflammatory mediators, leading to increased pulmonary capillary permeability and reduced gas exchange efficiency (9). Concurrently, CPB-related complications, such as low cardiac output syndrome, can indirectly impede respiratory recovery. Therefore, optimizing CPB management strategies—including modified priming solutions and efforts to minimize bypass time—holds significant clinical importance for mitigating its negative impact on respiratory function (10). Notably, the difference in postoperative mechanical ventilation duration cannot be solely attributed to CPB intervention, as evidenced by: 1. Airway Factors: Three patients in the complex group had pre-existing severe tracheobronchial structural abnormalities (tracheal stenosis, laryngomalacia), directly contributing to prolonged postoperative mechanical ventilation. 2. Multifactorial Prognostic Influence: The single mortality occurred in an infant with concomitant TOF, severe malnutrition, and critical airway pathology. The death, resulting from respiratory failure due to residual laryngomalacia 10 days post-discharge, highlights the profound impact of underlying comorbidities on long-term prognosis.

Accurate and timely diagnosis of DAA relies on imaging studies. In contrast, echocardiography is cost-effective and noninvasive, making it the preferred initial diagnostic tool (11). However, its limitations include difficulty in evaluating tracheal or esophageal compression and missing nonperfused vascular segments (12, 13). CTA provides superior visualization of aortic arch morphology and sites of compression, aiding in both diagnosis and surgical planning (14). In our cohort, CTA had a diagnostic accuracy of 100%, compared with 80% for echocardiography, consistent with previous reports (15). Notably, two cases in our study with intracardiac anomalies were misdiagnosed by echocardiography but correctly identified using CTA, underscoring the complementary role of these modalities. Combining echocardiography and CTA improves diagnostic accuracy and is recommended for comprehensive preoperative assessment (16).

No consensus exists regarding the optimal timing of DAA intervention. Some studies suggest monitoring asymptomatic or mildly symptomatic cases with regular follow-up (10), while others recommend early surgical correction regardless of symptom severity (11, 17–20). Most current studies favor early intervention to relieve tracheal and esophageal compression and prevent complications such as chronic hypoxia or respiratory arrest. Consistent with prior research, our findings indicate that symptoms improve with age as the trachea matures after compression is alleviated (13, 21).

One patient in the present study required reintubation postoperatively, with this requirement potentially being related to preexisting tracheal compression and subsequent tracheal softening postsurgery, which were exacerbated by laryngeal edema from intubation. Our findings suggest that careful surgical planning, including full mobilization of the aorta and minimizing scar formation, can reduce the need for reoperations, which is consistent with the experience of other centers (22, 23).

The long-term prognosis for patients DAA is generally favorable. However, a study on the management of DAA and associated outcomes in 81 patients revealed that long-term postoperative mortality was associated with persistent tracheal stenosis or tracheomalacia caused by continued compression (24). Given the critical role of tracheal malformation in patient outcomes, surgeons must give careful attention to tracheal status. Although tracheal stenosis often represents a secondary consequence of DAA, gradual improvement can occur following surgical decompression (5). This study has several limitations. First, it was a single center retrospective study with a small sample size and extended data collection period, which may have influenced the generalizability of the findings. Additionally, variations in the surgical techniques employed by different surgeons could introduce biases. Further prospective, multicenter studies with larger sample sizes are warranted to comprehensively assess long-term outcomes and treatment efficacy.

## 5 Conclusion

Surgical intervention and perioperative management for DAA in infants are safe and effective, yielding favorable clinical outcomes, specifically in cases of DAA with intracardiac anomaly. Early and accurate diagnosis, coupled with timely surgical correction, is pivotal in improving patient prognosis and quality of life. Comprehensive preoperative assessment, advances in surgical techniques, effective extracorporeal circulation management, and meticulous perioperative care contribute to successful treatment. Although this study provides valuable insights into DAA treatment, further large-scale, prospective investigations are necessary to refine treatment protocols and enhance long-term outcomes.

## Data Availability

The raw data supporting the conclusions of this article will be made available by the authors, without undue reservation.
